# Glutaredoxin 3 promotes nasopharyngeal carcinoma growth and metastasis *via* EGFR/Akt pathway and independent of ROS

**DOI:** 10.18632/oncotarget.9454

**Published:** 2016-05-18

**Authors:** Feng He, Lili Wei, Wenqi Luo, Zhipeng Liao, Bo Li, Xiaoying Zhou, Xue Xiao, Jingping You, Yufeng Chen, Shixing Zheng, Ping Li, Mariko Murata, Guangwu Huang, Zhe Zhang

**Affiliations:** ^1^ Department of Otolaryngology-Head and Neck Surgery, First Affiliated Hospital of Guangxi Medical University, Nanning, China; ^2^ Department of Pathology, First Affiliated Hospital of Guangxi Medical University, Nanning, China; ^3^ Department of Environmental and Molecular Medicine, Mie University Graduate School of Medicine, Mie, Japan

**Keywords:** glutaredoxin 3, nasopharyngeal carcinoma, EGFR, Akt

## Abstract

Glutaredoxin 3 (GLRX3) is antioxidant enzyme, maintaining a low level of ROS, thus contributing to the survival and metastasis of several types of cancer. However, the expression and functions of GLRX3 have not been addressed in nasopharyngeal carcinoma (NPC). In this study, we found that GLRX3 was overexpressed in NPC. Knockdown of GLRX3 in NPC cell lines inhibited proliferation *in vitro*, tumorignesis *in vivo*, and colony formation. In addition, GLRX3 knockdown decreased the migration and invasion capacity of NPC cells by reversing the epithelial-mesenchymal transition (EMT). Furthermore, stabilization of GLRX3 was positively related to with epidermal growth factor receptor (EGFR) expression and negatively with ROS generation. Phosphorylation of Akt, a key downstream effector, was induced by EGFR signaling but did not rely on increasing ROS level in NPC cells. GLRX3 might be an oncoprotein in NPC, playing important roles in increasing redox reaction and activating EGFR/ Akt signals, so it may be a therapeutic target for NPC.

## INTRODUCTION

Nasopharyngeal carcinoma (NPC) is a kind of head and neck cancer, rare throughout most of the world but frequently occurs in certain geographic areas, such as Southeast Asia and southern China. Multiple factors are involved in the carcinogenesis of NPC, including genetic susceptibility, environmental factors and Epstein-Barr virus (EBV) infection [1]. NPC is conventionally treated with radiotherapy. Irradiation exposure can cause DNA damage and mitochondrial-dependent reactive oxygen species (ROS) generation, critical mediators of radiation-induced cellular toxicity [2, 3]. In this context, the ROS level within NPC cells might be highly relevant to the therapeutic response. Although early-stage NPC can be cured by radiotherapy, a significant number of patients still show local recurrence and distant metastases, which highlights the need for a better understanding of the molecular mechanisms underlying therapeutic failure and developing an effective strategy for NPC therapy [4]. Exploring ROS-associated signaling in cancer cells might be a promising approach.

Over the past few decades, a number of studies have demonstrated that ROS are involved in cancer development by initiating and maintaining the oncogenic phenotypes of cancer cells [5]. Oxidative stress was observed in tumor biopsies and in the blood of NPC patients, so long-standing oxidative stress could be a pathogenic factor in NPC development [6]. Oxidative stress in the nasopharyngeal area might be caused by EBV latent infection, because the infection is associated with the production of ROS in NPC and other EBV-associated diseases [7–9]. Cellular antioxidative defense mechanisms for counteracting the excessive ROS and protecting against oxidative stress are also important for cell survival. For example, the only viral nuclear protein expressed in NPC, EBV nuclear antigen 1 (EBNA1), has been shown to induce ROS level in NPC cells. However, EBNA1 induces the antioxidants superoxide dismutase 1 and peroxiredoxin 1. Furthermore, ROS induction is not a prerequisite for EBNA1-mediated antioxidants, so these two effects may occur independently [9]. Therefore, the interplay between the counterbalancing effects of ROS and antioxidative defense may be important for NPC development and progression.

Cellular redox homeostasis is maintained by several intracellular redox-regulating molecules, including the thioredoxins (Trxr) and glutathione (GSH)-glutaredoxin (Grxr) system. Glutaredoxin 3 (GLRX3), also known as TXNL2, Grx3 and PICOT, is a multi-domain protein that contains two N-terminal monothiol Grx domains and an additional C-terminal Trx domain. GLRX3 is conserved in eukaryotes [10]. Deletion of GLRX3 in mice causes embryonic lethality, so GLRX3 is essential in protecting cells against oxidative stress during embryogenesis [11]. GLRX3 was characterized as an iron-sulfur protein and as a redox sensor in signal transduction in response to redox signals by reactive oxygen and nitrogen species [12], thus playing a key role in celluar signal transduction in response to stress signals by ROS [10]. Transient overexpression of GLRX3 attenuated the activation of c-Jun N-terminal kinase and transcription factors AP-1 and NF-κB in Jurkat T cells [13]. GLRX3 has broad interaction with other molecules modulating major cellular pathways.

Overexpression of GLRX3 was observed in human malignancies such as hepatocellular carcinoma, lung and breast cancer [14–16]. Knockdown of GLRX3 in human breast cancer cells reduced NF-κB activity, thereby inhibiting *in vitro* proliferation, survival, and invasion [15]. GLRX3 modulates redox-signaling pathways that contribute to malignant transformation [17], and GLRX3 itself also affects multiple cellular pathways. The complicated roles of GLRX3 in cancer have remained largely undiscovered.

From our understanding of the interaction of GLRX3 and the redox signaling in cancer cells, we hypothesized that GLRX3 may be an important molecule in NPC development and progression. We assessed GLRX3 expression in NPC cells and primary NPC tissues, investigated the biological function of GLRX3, and studied the associated signaling events.

## RESULTS

### GLRX3 is overexpressed in NPC

**Figure 1:**
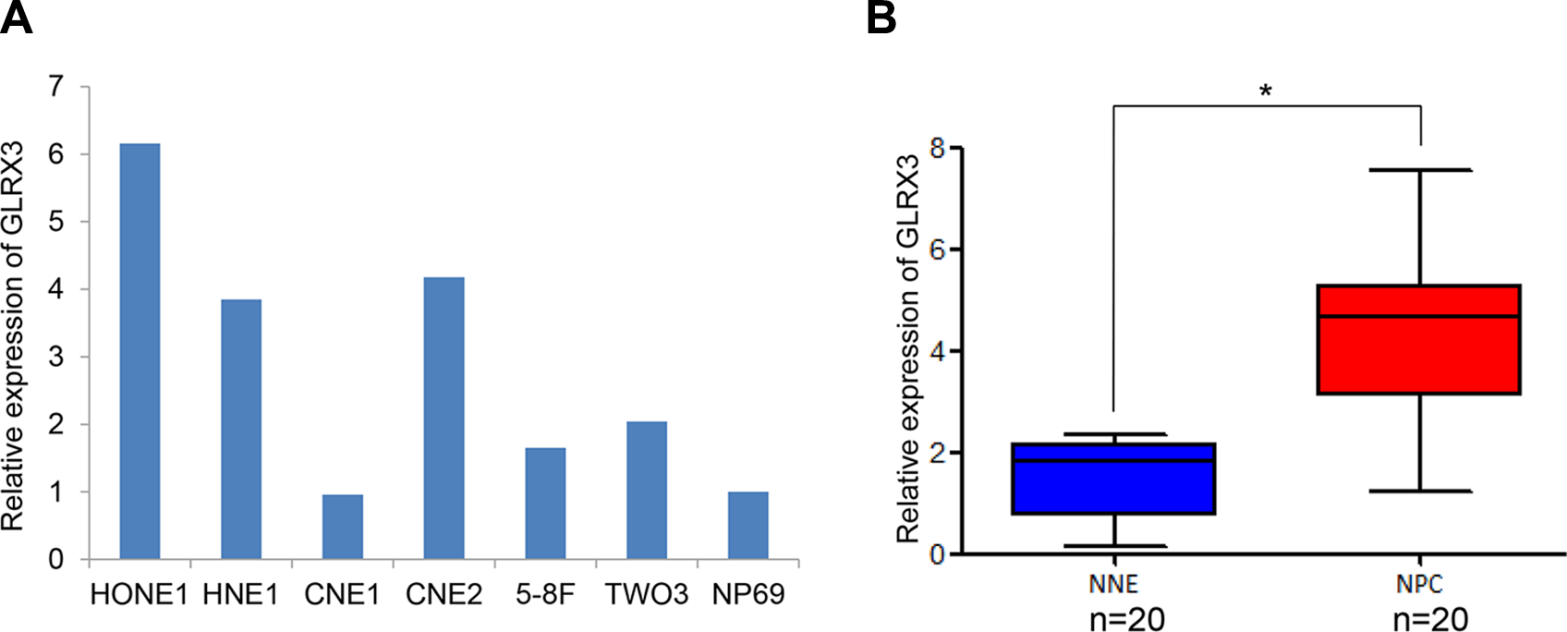
mRNA level of *GLRX3* in nasopharyngeal carcinoma (NPC) and normal nasopharyngeal epithelia (NNE) (**A**) Real-time PCR of the mRNA level of *GLRX3* in 6 NPC cell lines and a non-cancerous nasopharyngeal epithelial cell line NP69. (**B**) Relative *GLRX3* mRNA expression in NPC primary biopsies (*n* = 20) and NNE samples (*n* = 20). Boxes indicate 25 to 75 percentile, horizontal line indicates the mean, and bars indicate 10 and 90 percentile (*p < 0.05).

We assessed the transcription of *GLRX3* in six NPC cell lines, HONE1, HNE1, CNE1, CNE2, 5-8F, TW03, and a non-malignant human nasopharyngeal epithelial cell line, NP69. Except for CNE1 cells, most of the NPC cell lines showed a higher mRNA level of *GLRX3* as compared with NP69 cells (Figure 1A). Also, the mRNA level of *GLRX3* was greater in NPC tissues (*n* = 20) than normal control tissues (*n* = 20) (Figure 1B).

**Figure 2:**
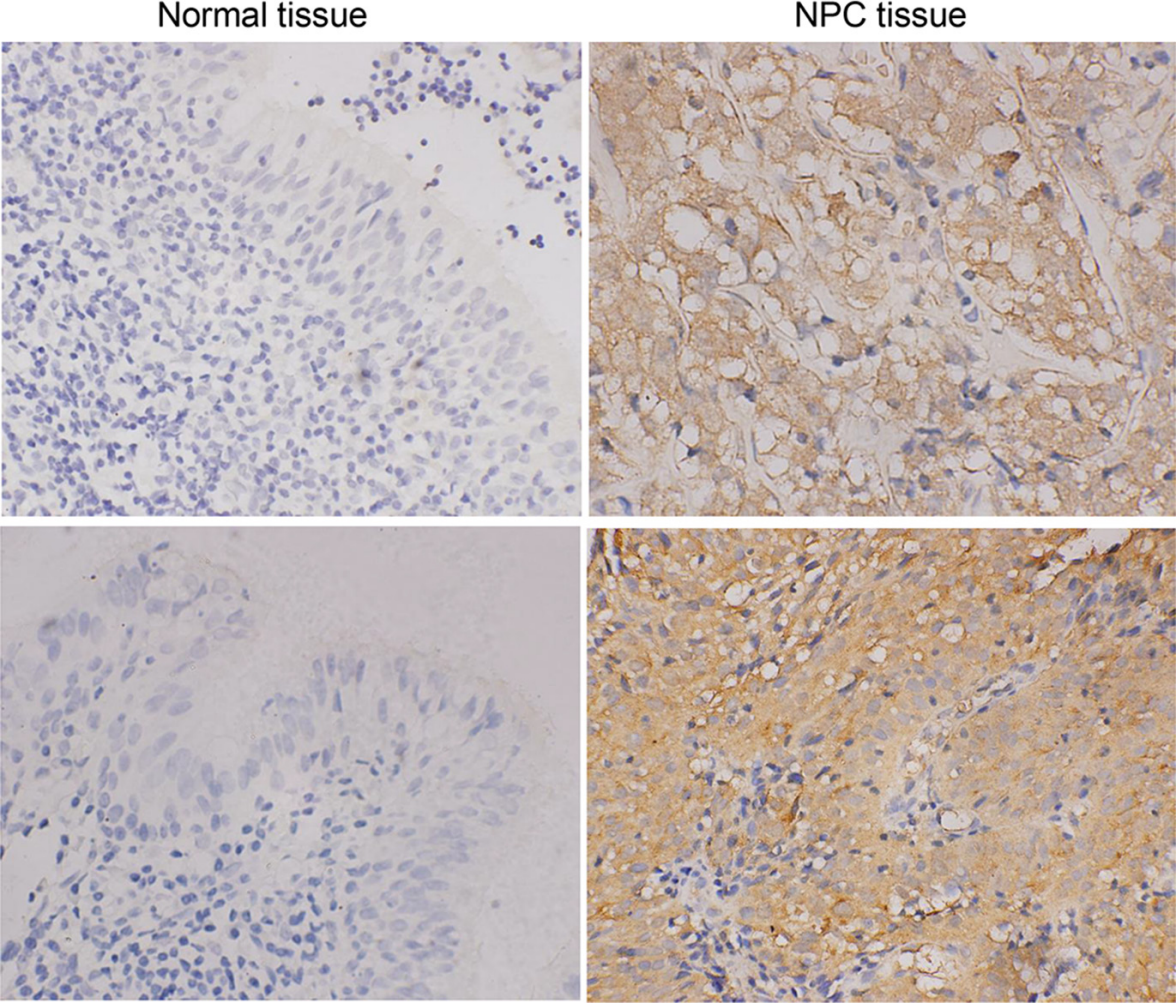
Immunohistochemical staining of GLRX3 protein expression in NPC (*n* = 59) and NNE tissue (*n* = 30) Magnification ×400.

Next, we analyzed GLRX3 protein expression in 59 cases of NPC tissues and 30 cases of normal tissues. GLRX3 was localized in the cytoplasm of NPC cells (Figure 2). Overall, 37 of 59 (62.7%) NPC tissues showed strong expression of GLRX3, whereas only 11 of 30 (36.7%) non-cancerous control samples showed positive GLRX3 expression. The difference between NPC tissues and the controls was significant (Table 1). Furthermore, GLRX3 protein expression was not associated with clinical parameters of NPC patients, including gender, age, histological type, clinical stage, T and N classification, and distant metastasis status (Table 2).

**Table 1:** GLRX3 expression in nasopharyngeal carcinoma (NPC) tissues and normal tissues

Group	+	−	*P* value
NPC tissues (*n* = 59)	37 (62.7%)	22 (37.3%)	0.025*
Normal tissues (*n* = 30)	11 (36.7%)	19 (63.3%)	

GLRX3-negative staining and -positive staining are indicated by “–” and “+”, respectively.

**Table 2:** The correlation between the clinical characteristics and GLRX3 expression in NPC patients

	Cases	GLRX3		*P* value^a^
		+	−	
Gender				
Male	44	29 (78.3%)	15 (68.2%)	NS
Female	15	8 (21.7%)	7 (31.8%)	
Age (y)				
< 40	21	12 (32.4%)	9 (40.9%)	NS
40–50	15	10 (27.0%)	5 (22.7%)	
≥ 50	23	15 (40.6%)	8 (36.4%)	
Non-keratinizing carcinoma				
Undifferentiated	54	31 (91.2%)	23 (92.5%)	NS
Differentiated	5	3 (8.8%)	2 (7.5%)	
Clinical stage^b^				
I, II	1	1 (2.7%)	0 (0.0%)	NS
III, IV	58	36 (97.3%)	22 (100%)	
Lymph node metastasis				
+	55	34 (91.9%)	21 (95.5%)	NS
–	4	3 (8.1%)	1 (4.5%)	

a by Pearson chi-square test or Fisher's exact test.

b according to the International Union Against Cancer (UICC).

NS: not significant.

### Knockdown of GLRX3 inhibits NPC cell growth both *in vitro* and *in vivo*

**Figure 3:**
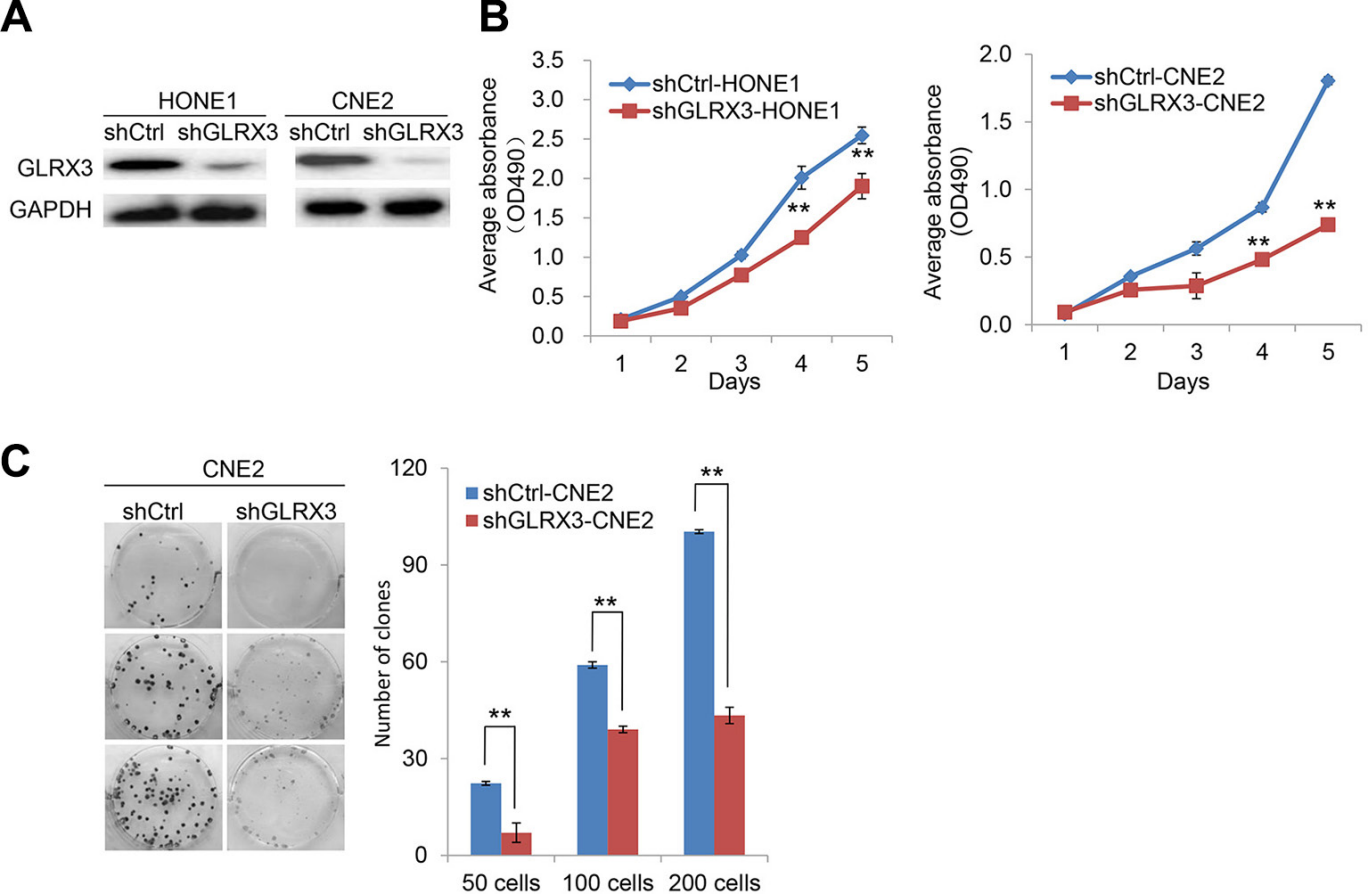
Knockdown of GLRX3 suppresses the growth of NPC cell line *in vitro* (**A**) Western blot confirmation of the silencing effect of *shGLRX3* construct in HONE1 and CNE2 cell lines. (**B**) MTT assay of growth curves of sh*GLRX3*-HONE1 and *shGLRX3*-CNE2 and their *shCtrl* cell line. Data are mean ± SD of five independent experiments. (**C**) Representative colony images and quantification of colonies in CNE2 cells with and without GLRX3 knockdown. Data are mean ± SD of three independent experiments (**p < 0.01).

**Figure 4:**
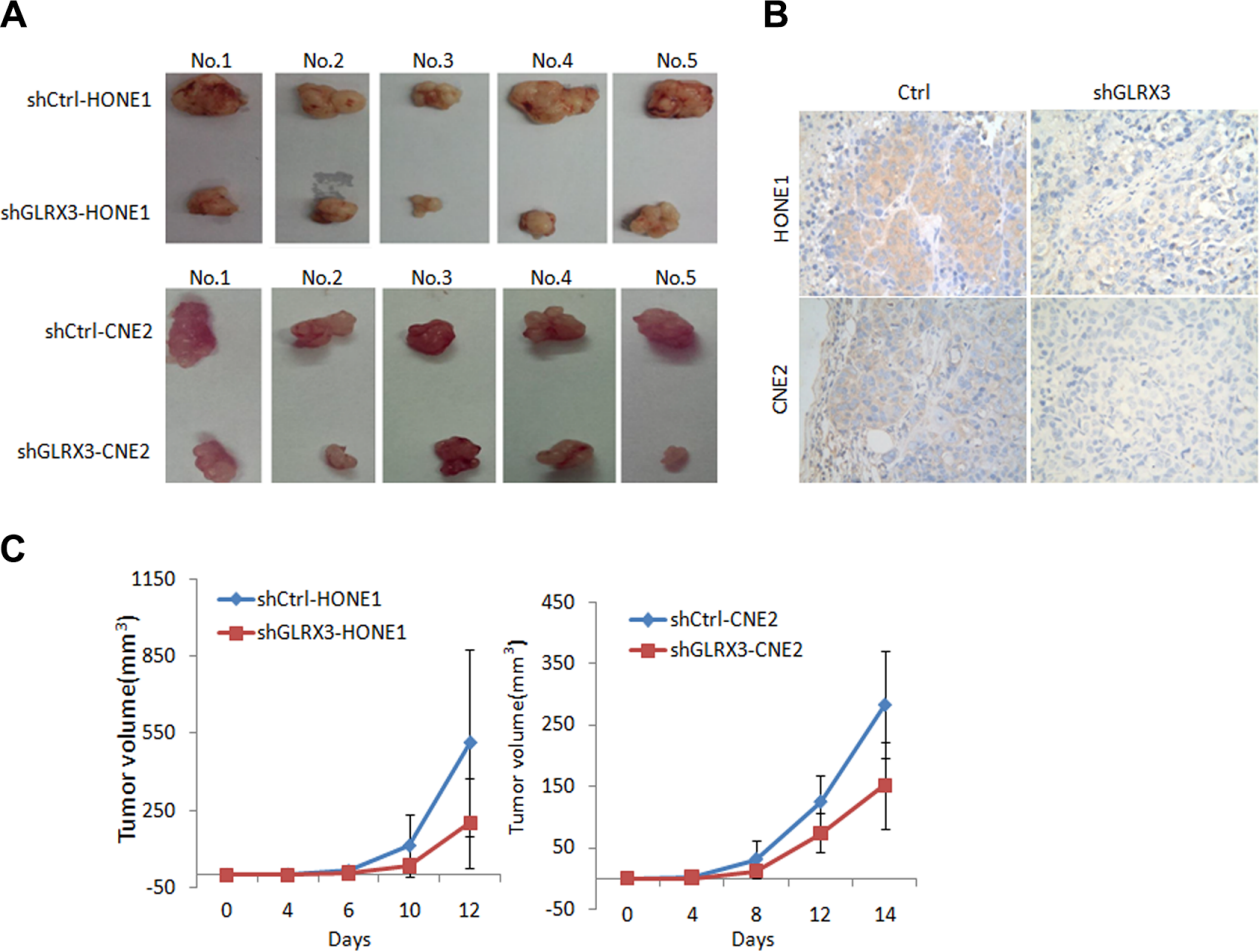
Knockdown of GLRX3 reduced tumorigenicity of NPC cells in nude mice (**A**) Tumors from nude mice 2 weeks after inoculation. (**B**) Immunohistochemical staining of GLRX3 protein expression in tumors from nude mice. Magnification ×400. (**C**) Growth curves of tumors derived from HONE1 and CNE2 cells with and without *shGLRX3* in nude mice. The volume of tumors was measured every 2 days after inoculation. Data are mean ± SD from five experiments.

To investigate the biological function of GLRX3 in NPC, we established HONE1 and CNE2 cell lines with stable knockdown of GLRX3 (*shGLRX3*-HONE1/CNE2) and corresponding control cell lines (*shCtrl*-HONE1/CNE2) (Figure 3A). MTT assay was used to assess the effects of GLRX3 on NPC cell proliferation. The growth of *shGLRX3* NPC cells was suppressed as compared with control cells (Figure 3B). Transiently overexpressed *GLRX3* in CNE1, with relatively low expression of *GLRX3*, promoted cell proliferation (Supplementary Figure S1). *shGLRX3*-CNE2 cells showed fewer colonies than control cells on colony-formation assay (Figure 3C). Furthermore, we evaluated the tumorigenicity of *shGLRX3*-HONE1 and *shGLRX3*-CNE2 cells and their control cell lines *in vivo*. All cells developed a tumor mass in nude mice about 2 weeks after inoculation, but the size of tumors from *shGLRX3*-HONE1 and *shGLRX3*-CNE2 cells was smaller than control cell lines (Figure 4A). We confirmed again the GLRX3 expression remains lower in the tumors derived from *shGLRX3*-HONE1 and *shGLRX3*-CNE2 cells (Figure 4B). As well, tumors from *sh*GLRX3-HONE1/CNE2 NPC cells showed slower growth (Figure 4C). Therefore, knockdown of GLRX3 inhibited the proliferation of NPC cells, both *in vitro* and *in vivo*.

### Knockdown of GLRX3 inhibits cell invasion and migration by reversing the EMT

**Figure 5:**
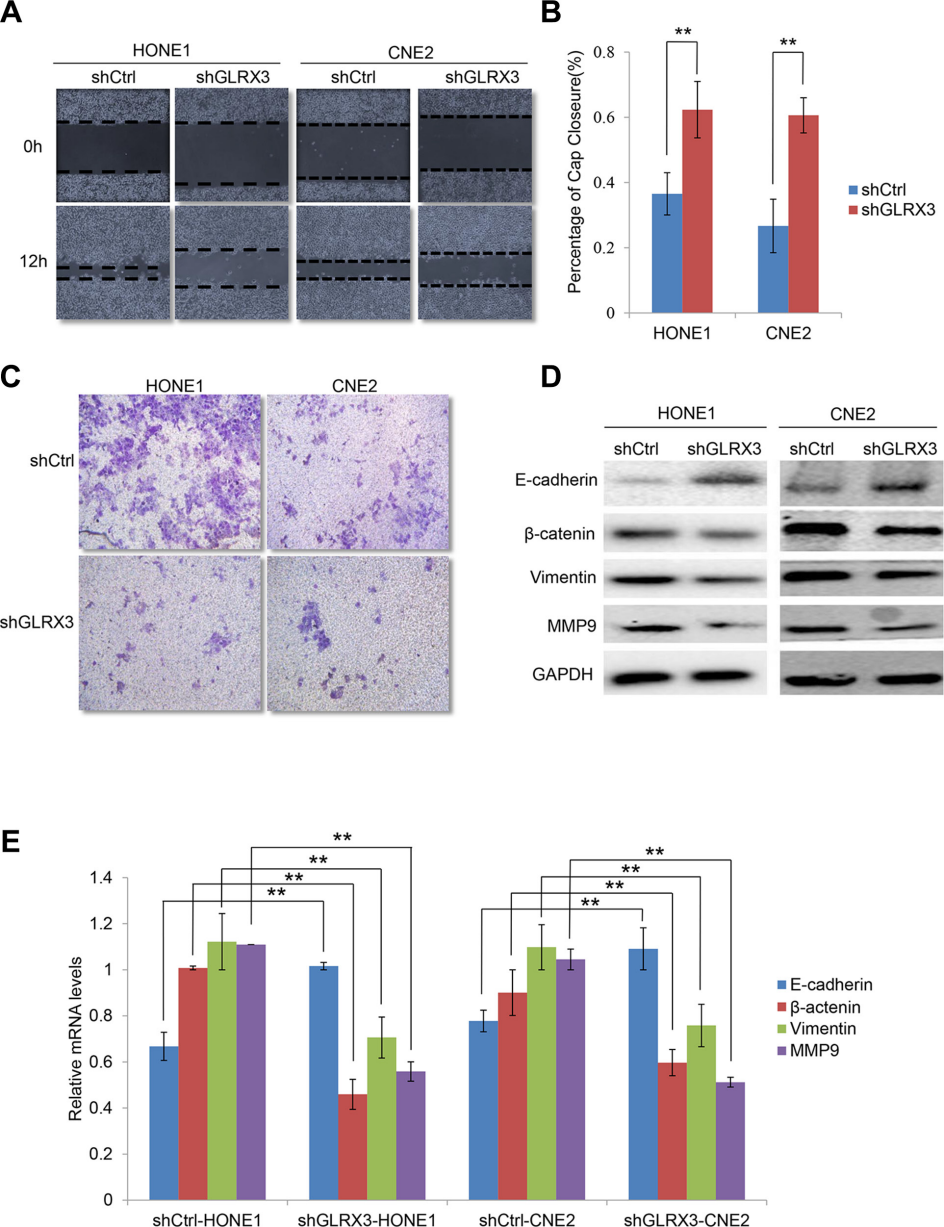
Knockdown of GLRX3 inhibits migration and invasion of NPC cells (**A**) Wound-healing assay. Images were taken at 0 and 12 h after introducing a scratch in *shGLRX3*-HONE1/CNE2 and shCtrl-HONE1/CNE2 cells. (**B**) Gap closure is measured as mean ± SD of three independent experiments. (**C**) Transwell invasion assay of invasive capacity of HONE1 and CNE2 cells. The violet color dots represent cells penetrating through matrix gels. (**E**) Real-time RT-PCR assay of *E-cadherin, β-catenin, Vimentin* and *MMP9* mRNA levels (**D**) and western blot assay of protein levels (E). Data are mean ± SD from three experiments. (**p < 0.01)

GLRX3 promotes the motility of breast and colon cancer cells [15, 18]. Thus, we investigated the effect of GLRX3 on migration and invasion of NPC cells. Wound healing assay revealed slower gap closure in *shGLRX3*-HONE1 and *shGLRX3*-CNE2 cells with a significant decrease in cell migration ability (Figure 5A, 5B). On Transwell assay, *shGLRX3*-HONE1 and *shGLRX3*-CNE2 cells showed reduced capacity for invasion during overnight culture (Figure 5C).

To further evaluate whether GRLX3 affected migration and invasion via reversing the EMT, we analyzed the expression of *E-cadherin, β-catenin, Vimentin* and *MMP9* at mRNA and protein levels. In *shGLRX3*-HONE1 and *shGLRX3*-CNE2 cells, the mRNA level of *E-cadherin* was upregulated in knockdown cells, whereas that of β-catenin, Vimentin and *MMP-9* was downregulated (Figure 5D–5E). Thus, GLRX3 may be involved in the EMT process of NPC cell lines. Overexpression of GLRX3 may increase the risk of invasion and metastasis in NPC patients by inducing the EMT.

### Knockdown of GLRX3 contributes to inactivation of Akt signaling independent of ROS in NPC cells

**Figure 6:**
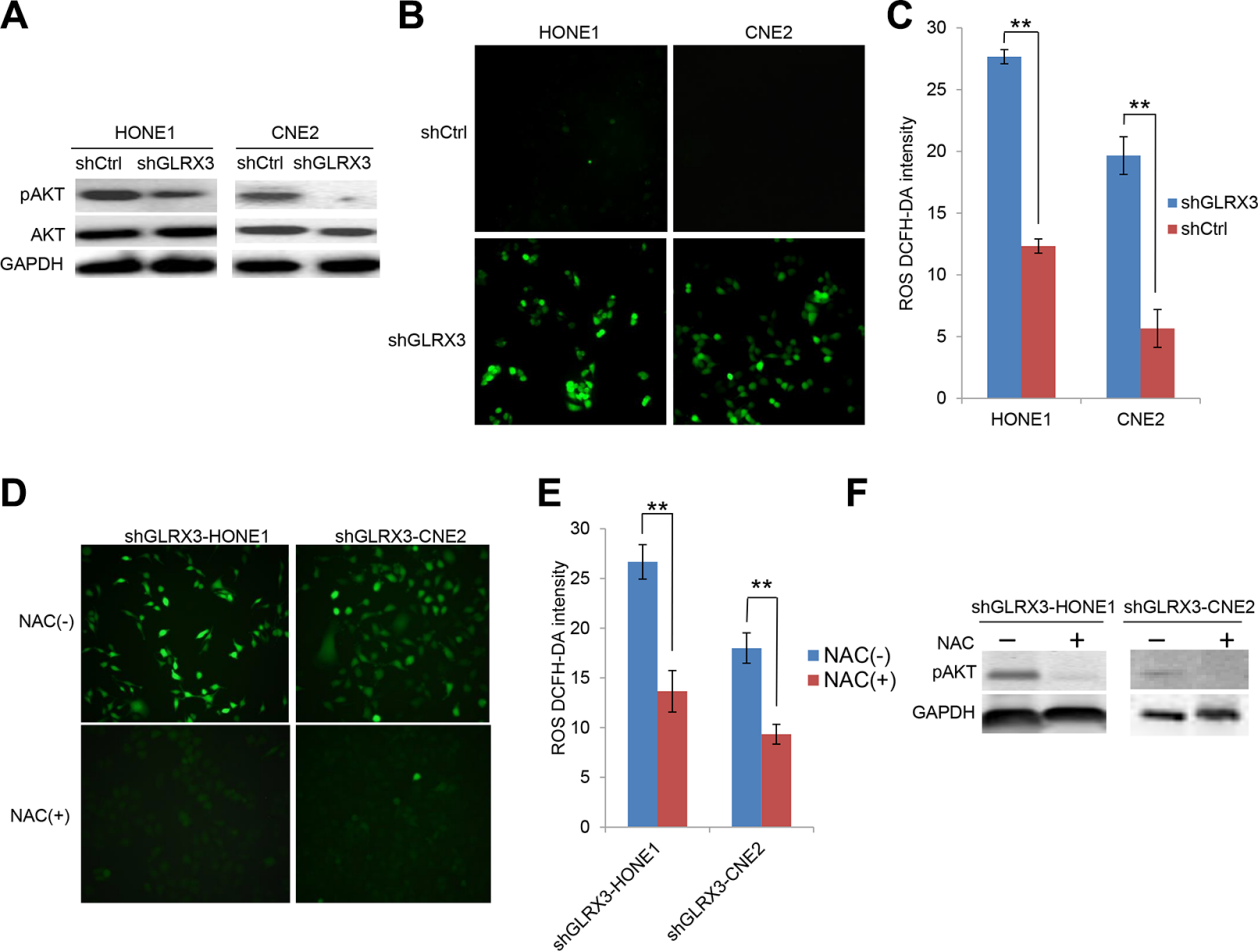
Knockdown of GLRX3 in NPC cells decreases pAKT independent of reactive oxygen species (ROS) generation (**A**) Western blot analysis of protein levels of pAkt and Akt in shGLRX3-HONE1/CNE2 cell lines. (**B**) ROS were detected by DCFH-DA staining with green fluorescent signal (magnification ×200). (**C**) The intensity of ROS was calculated as relative light unit. Data are mean±SD from three experiments (**D–E**) ROS detection in shGLRX3-HONE1 and shGLRX3-CNE2 cells treated with and without ROS inhibitor 5 mM NAC for 48 h, followed by intensity analysis. (**F**) Western blot analysis of pAkt protein level. Data are mean ± SD from three experiments. (**p < 0.01)

The PI3K/Akt pathway is instrumental in proliferation, EMT and angiogenesis during tumorigenesis [19]. Recent study has shown that GLRX3 interacts with the PI3K/Akt pathway to promote the motility of colon cancer cells [18]. Here, we found that phosphorylation of Akt was markedly suppressed in *shGLRX3*-HONE1 and *shGLRX3*-CNE2 cells (Figure 6A). As an antioxidant molecule, GLRX3 may affect the intracellular redox system. We found that ROS generation was significantly elevated in NPC cells when GLRX3 was knocked down (Figure 6B, 6C). The effect of ROS on activating the Akt pathway remains controversial [20, 21]. To identify whether the hypergeneration of ROS by silencing GLRX3 in NPC cell lines contributes to inhibiting the Akt signaling cascade, we decreased the ROS level in *shGLRX3*-HONE1/CNE2 cells by an ROS inhibitor N-acetyl cysteine (NAC) (Figure 6D, 6E). However, we found no reactivation of Akt but rather even more downregulation of pAkt (Figure 6F), so GLRX3 exerts its function in suppressing pAkt independent of ROS.

### Knockdown of GLRX3 is associated with downregulation of epidermal growth factor receptor (EGFR), a key upstream effecter of Akt signaling

**Figure 7:**
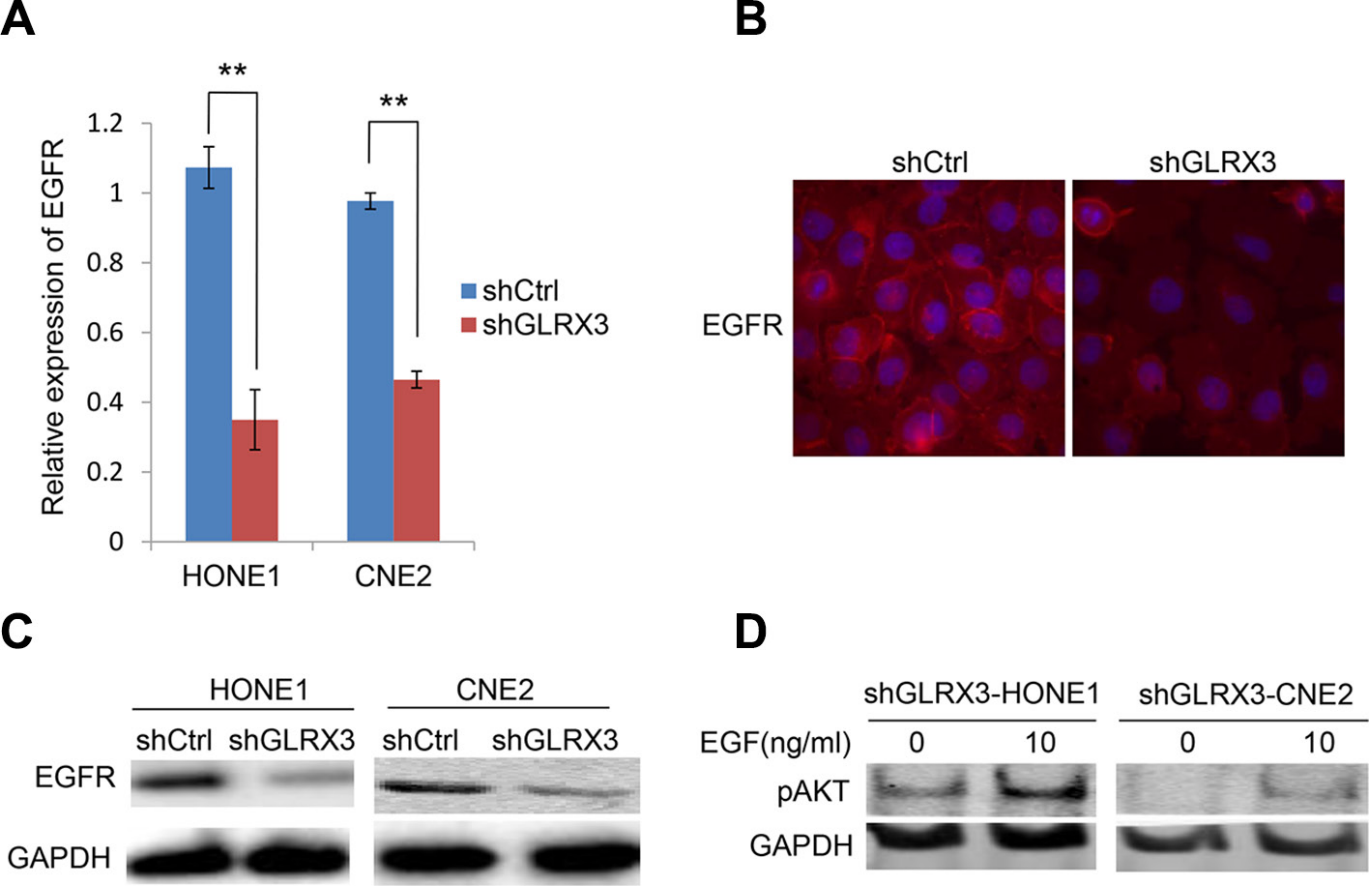
Epidermal growth factor receptor (EGFR) is essential for the effects of GLRX3 on inhibiting pAkt (**A–C**) Real-time RT-PCR assay of mRNA levels of EGFR in shGLRX3-HONE1/CNE2 and shCtrl-HONE1/CNE2 cells (A), and western blot (B) and immunofluorescence (C) assay of protein levels. Data are mean ± SD from three experiments. (**D**) pAkt detection in shGLRX3-HONE1 and shGLRX3-CNE2 cells treated with and without EGFR stimulator 100 ng/ml EGF for 48 h, followed by western blot analysis. (**p < 0.01)

The PI3K/Akt pathway is downstream of EGFR and is emerging as possibly one of the most important pathways in head and neck squamous cancers [22, 23]. Therefore, we assessed the expression of EGFR at both the mRNA and protein levels. EGFR level was impaired in *shGLRX3*-HONE1 and sh*GLRX3*-CNE2 cells (Figure 7A–7C). Ectopic expression of *GLRX3* in CNE1 cells upregulated the expression of EGFR (Supplementary Figure S2). Then, to identify the possible association of EGFR and pAkt levels, we treated cells with GLRX3 knockdown with the EGFR signaling stimulator EGF to activate the lower but remaining EGFR level. Akt was activated after stimulation (Figure 7D). Therefore, the effect of GLRX3 on dephosphorylation of Akt might due to impaired EGFR expression instead of ROS generation.

## DISCUSSION

GLRX3 is overexpressed in several human cancers [15, 16, 18]. In agreement, we found both the transcription and protein levels of GLRX3 elevated in NPC cell lines and primary tumors. Knockdown of GLRX3 inhibited NPC cell proliferation *in vitro* and *in vivo* and also colony formation, cell migration and invasion by reversing the EMT. GLRX3 might be a putative oncogene modulating tumor growth and metastasis in NPC.

In normal cells, low to moderate levels of ROS are essential for cellular proliferation, differentiation and survival [24]. In contrast, excessive ROS results in cellular toxicity and induces apoptosis [25, 26]. Oxidative stress, resulting from an imbalance between the generation and scavenging of ROS, may be involved in the whole process of tumorigenesis and progression [27]. ROS dysregulates the cellular redox homeostasis and initiates tumor formation by damaging both nuclear DNA and mitochondrial DNA and triggering an aberrant cascade of signaling networks [28, 29]. During cancer progression, tumor cells show enhanced oxidative status due to their high metabolic rate [30]. Tumor cells start or initiate a strong antioxidative defense mechanism to counterbalance the excessive ROS, thus suppressing cell apoptosis, and become highly malignant with drug resistance or become cancer stem cells [31, 32]. Evidence has highlighted a link between the EMT and cancer stem cells that initiate and maintain tumors. EMT has been implicated in cancer cell invasion. Overexpression of GLRX3 might induce the EMT and also inhibit the cellular ROS level. GLRX3 might be essential to maintain a metastatic status.

In NPC, a low ROS level might be essential for maintaining EBV infection. EBV exists in every tumor cell in the form of latent infection in NPC tissues. More than 120 genes are encoded by EBV, but only a few are expressed in NPC latent infection. By this means, the infected cells thus escape from immune attack by reduced immunogenicity. A recent study reported that increased ROS production can trigger the reactivation of EBV from latency in NPC cells [33]. Consistent with this finding, we found overexpression of GLRX3 in NPC, and its knockdown increased the production of intracellular ROS, so intracellular ROS is a major downstream effect of GLRX3. Hence, GLRX3 overexpression may be involved in the modulation of EBV latent infection in NPC cells, thus contributing to tumorigenesis and progression by reduced immunogenicity of NPC cells. This association of EBV latency and GLRX3 expression needs further investigation.

The stabilization of GLRX3 level affects several major pathways in human cancer, such as NF-κB [15], JNK signaling and FcepsilonRI-mediated pathway [34]. Our study supports this notion by revealing the ability of GLRX3 to trigger the phosphorylation of Akt, a key signal regulating cellular biological behavior, including cell growth and survival [35]. In addition, activation of Akt can induce the EMT, thereby endowing cancer cells with an invasive phenotype [35]. In NPC, phosphorylation of Akt is frequently observed and is related to the malignant phenotype [36–38]. EBV encodes latent membrane protein 1 (LMP1), which contributes to loss of function of PTEN, an inhibitor of the PI3K/Akt pathway, by upregulating miR-21, thus potentiating NPC [39] and promoting chemoresistance [40]. In addition, LMP1 stimulates anaerobic glycolysis by activating Akt [30]. Another EBV-encoded product, miR-BART7-3p, accelerates the NPC cell cycle depending on the PTEN/PI3K/Akt pathway [41]. However, the inhibitors of this important pathway could be impaired by epigenetic alterations in NPC, such as DNA methylation or aberrant miRNA expression [42, 43]. Here, we investigated an association between stabilization of GLRX3 and induction of pAkt. Hence, GLRX3 may promote cancer cell proliferation, invasion and the EMT via a PI3K/Akt pathway in NPC.

Inhibition of PI3K/Akt signaling can enhance the radiosensitivity of NPC cells [44, 45], whereas ROS are involved in NPC radiotherapy. Clearing ROS attenuates the activity of Akt, so ROS activity may serve as an important signal for Akt phosphorylation [20]. The effect of GLRX3 on cell activation and signal transduction may be induced by redox ROS [46]. However, GLRX3 lacks enzymatic activity [47], so it may function in cell signaling by mechanisms other than ROS. To validate this, we inhibited the ROS generation with an antioxidant NAC in GLRX3-knocked down NPC cells and found that the phosphorylation of Akt could not be reversed. Thus, Akt activation induced by GLRX3 is ROS-independent.

EGFR is a pivotal upstream modulator protein of the PI3K/Akt pathway. Inhibition of the EGFR/Akt pathway induces cellular senescence and suppresses the cancer stem cell phenotype in NPC cells [48, 49]. We found that knockdown of GLRX3 in NPC cells significantly downregulated EGFR expression at both the mRNA and protein levels, whereas EGF could slightly increase the pAkt level, which suggests that loss of EGFR expression accounts for lowering pAkt and suppressing cell proliferation, colony formation and metastasis capacity. Thus, GLRX3 modulates the EGFR/Akt pathway on cellular ROS signaling.

In summary, our study shows that GLRX3 is overexpressed in NPC cells and tumor tissues. Knockdown of GLRX3 inhibited cell proliferation, suppressed the EMT and inhibited NPC cell invasion and migration via EGFR/Akt signaling. GLRX3 inhibits the cellular ROS level, which might contribute to maintaining EBV latent infection and the EMT phenotype in NPC. These findings implicate an essential role for GLRX3 in NPC pathogenesis and suggest that GLRX3 is a novel pharmacology target for the treatment and prevention of NPC.

## MATERIALS AND METHODS

### Ethics statement

Ethical permission for this study was granted by the Research Ethics Committee of the First Affiliated Hospital of Guangxi Medical University (Nanning, China).

### Study samples

NPC derived cell lines HONE1, HNE1, CNE1, CNE2, 5-8F, and TW03 [50–53] were maintained in IMDM medium (Invitrogen, Carlsbad, CA, USA) containing 10% fetal calf serum (Invitrogen, Carlsbad, CA, USA) at 37°C in an atmosphere of 5% CO_2_. The nonmalignant nasopharyngeal epithelial cell line NP69 [54] was cultivated in defined keratinocyte-serum free medium.

In total, 90 biopsies with NPC diagnosed by experienced pathologists according to the World Health Organization classification were collected from donors in the Department of Otolaryngology-Head and Neck Surgery, First Affiliated Hospital of Guangxi Medical University (Nanning, China). A total of 39 normal nasopharyngeal epithelial biopsy tissues were obtained from patients with chronic inflammation of the nasopharyngeal area as controls. Tissue from 20 patients with NPC and 20 controls was used for RNA extraction. The remaining biopsies (including 59 samples of NPC tissue and 30 of normal tissues) were fixed in 10% par formaldehyde embedded in paraffin, and cut in 4-μm serial sections.

### Retroviral vector-mediated *GLRX3*-specific shRNA stable transfection

For silencing of *GLRX3* in HONE1 and CNE2 cells (*shGLRX3*-HONE1/CNE2, *shCtrl*-HONE1/CNE2), the sense- and antisense-strand oligonucleotides 5′-tcgag AAGATCTCAACCTTCGCTTGAttcaagagaTCAAGCGAA GGTTGAGATCTTtttttggaaaa-3′ and 5′-gatcttttccaaaaaAA GATCTCAACCTTCGCTTGAtctcttgaaT CAAGCGAAGG TTGAGATCTTc-3′, respectively, were used in a retroviral construct pBINNS2. Single, puromycin-resistant clones with the most efficient *GLRX3* downregulation were selected and used in this experiment. The efficacy of silencing *GLRX3* was confirmed by western blot analysis. A pool of single cell clones transformed with scramble *shRNA* was used as an empty control.

### Quantitative real-time PCR

Total RNA was isolated by use of Trizol Reagent (Invitrogen, Carlsbad, CA, USA) and reverse transcribed by use of the Prime Script RT reagent kit (Invitrogen, Carlsbad, CA, USA). Quantitative real-time PCR involved use of Roche Fast Start Universal Green Master. The primer sequences and cycling conditions for all experiments are in Table 3. The mRNA expression was determined by the 2^−ΔΔC^ method.

**Table 3:** qPCR primer sequences

Primers	Sequences	Product size	Annealing temperature
***GLRX3***	Forward: 5′-CGCTGTGGTTTCAGCAAGC-3′	199 bp	60°C
	Reverse: 5′-CTTCAGATGCTTCTAGCTCCTT-3′		
***E-cadherin***	Forward: 5′-CGCCTTATGATTCTCTGCTCGTGTT-3′	130 bp	60°C
	Reverse: 5′-CGATTGCCCCATTCGTTCAAGTAGT-3′		
***β-catenin***	Forward: 5′-GCTGCTGTTTTGTTCCGAAT-3′	213 bp	60°C
	Reverse: 5′-CTGGCCATATCCACCAGAGT-3′		
***Vimentin***	Forward: 5′-TACATCGACAAGGTGCGCTT-3′	152 bp	60°C
	Reverse: 5′-TCGTTGGTTAGCTGGTCCAC-3′		
***MMP-9***	Forward: 5′-ACCTGTACCGCTATGGTTAC-3′	150 bp	60°C
	Reverse: 5′-GTGGGGTTCGCATGGCCTTC-3′		
***EGFR***	Forward: 5′-GTGAACCCCGAGGGCAAATA-3′	162 bp	60°C
	Reverse: 5′-AGGCCCTTCGCACTTCTTAC-3′		
***GAPDH***	Forward: 5′-GCACCGTCAAGGCTGAGAAC-3′	138 bp	60°C
	Reverse: 5′-TGGTGAAGACGCCAGTGGA-3′		

### Immunohistochemical analysis

First, tissue sections were blocked with 5% bovine serum albumin (BSA) for 1 h after deparaffinization and rehydration then antigen retrieval, then incubated with mouse anti-human GLRX3 antibody (Santa Cruz Biotechnology, Santa Cruz, CA, 1:100) at 4°C overnight, followed by goat anti-rabbit secondary antibody with 3, 3′-diaminobenzidine (DAB) reagent (ZSGB-BIO) with counterstaining with haematoxylin. Finally, images were acquired under a microscope (Olympus C-5050, Japan) and analyzed with use of Image-Pro Plus 6.0 (Media Cybernetics, USA).

### Cell proliferation assay

*shCtrl*-HONE1/CNE2 and *shGLRX3*-HONE1/CNE2 cells were seeded in 96-well plates at 2 × 10^3^ cells per well. Cell density was measured by using the vital stain 3-(4, 5-dimethylthiazol-2-yl)-2, 5-diphenyltetrazolium bromide (MTT, Solarbio) with absorbance at OD490 nm (iMark, Bio-Rad, USA) for 5 days. Each treatment was performed in quintuplicate.

### Colony formation assay

Cells were seeded in 6-well plates at 50, 100 and 200 cells/well. The medium was changed every 3 days. After 14 days, Giemsa-stained colonies were photographed and counted by use of Quantity One v4.4.0 (Bio-Rad, USA). The experiment was performed in triplicate.

### *In vivo* tumor growth assay

Five female and 6-week-old Balb/cathymic nude mice (Experimental Animal Center of Guangxi Medical University, China) were injected with 1.0 × 10^6^ *shGLRX3*-HONE/CNE2 cells in the right flank, and an equal amount of *shCtrl*-HONE1/CNE2 cells was injected into the left flank as a control. The tumor volume was assessed by 2D measurements every 2 days [55].

### Wound healing assay

Cells at 5.0 × 10^5^ per well were seeded on 6-well plates and allowed to adhere overnight in growth media containing 1% fetal calf serum (FCS) for up to 90% confluence. The monolayer cells were scratched by using a sterile 200-μl pipette tip. After 12 h, wound closure was evaluated by light microscopy (Olympus, Japan). The experiment was performed in triplicate.

### Transwell assay

Cells at 2.5 × 10^4^ resuspended in serum-free IMDM medium were plated into each upper chamber of Bio-Coat Invasion Chambers (BD, Bedford, MA) coated with Matrigel. IMDM medium with 10% FCS was added to the lower chamber as a chemoattractant. At 48 hr, non-invading cells were removed with a cotton-tipped swab. Migratory and invasive cells on the lower membrane surface were fixed in 1% paraformaldehyde, stained with crystal violet, and photographed.

### ROS detection

The intracellular ROS generation was analyzed with use of an ROS assay kit (GMS10016.2, Gemmed Scientifics, USA) according to the manufacturer's protocol. Cells were incubated with DCFH-DA probe at 1:1000 dilution in the culture medium and maintained at 37°C for 30 min, then washed with serum-free IMDM medium three times and visualized under fluorescent microscopy and measured by use of a Micro Fluorescence Reader with excitation at 490 nm (BIO-TEK Instruments).

### Western blot analysis

In brief, proteins from cells were obtained by use of RIPA lysis buffer (Beyotime, Jiangsu, China) containing cocktail (ROCHE complete Mini; EDTA-free). Equal amounts of protein were separated by 10% SDS-PAGE and transferred to nitrocellulose filter (NC) membranes (Millipore, USA), which were blocked with 5% milk for 1 h at room temperature (RT), then incubated with primary antibodies to GLRX3 (1:1000, sc-100601) and β-catenin (1:1000, sc-376841, both Santa Cruz Biotechnology); E-cadherin (1:1000 3195P), Vimentin (1:1000, 5741P), pAkt (1:1000, 4060P) and GAPDH (1:1000, 5174P, all CST); and MMP9 (1:1000, ab137867) and EGFR (1:1000, ab52894, both Abcam) at 4°C overnight, followed by the appropriate peroxidase-conjugated secondary antibodies (anti-rabbit/mouse, 1:10000, 926–32211/926–68070, Licor). Chemiluminescent signals were captured by a CCD camera in a ChemiDoc XRS (Bio-Rad) instrument with Image Lab software.

### Immunofluorescence staining

Cells were grown on cover slips overnight, then fixed in 4% paraformaldehyde at room temperature for 20 min, washed with phosphate buffered saline (PBS), and permeabilized with 0.5% Triton X-100 PBS for 10 min. After blocking for 1 h in 5% BSA in PBS, cells were incubated with EGFR (1:200, sc-120, Santa Cruz Biotechnology) antibody diluted in 5% BSA/PBS for 1 h at room temperature, followed by incubation with Alexa Fluor 568 Donkey Anti-Rabbit IgG antibody (A11034, Life Technologies, Carlsbad, CA) for another 1 h at room temperature. Cells were washed with PBS and mounted in Vector shield with DAPI (Vector Laboratories, Burlingame, CA). Finally, images were taken by using a LSM710 confocal microscope (Carl Zeiss, Jena, Germany) at ×200 magnification.

### Statistical analysis

All data were analyzed by using SPSS 16.0 (SPSS Inc., Chicago, IL, USA). Data are expressed as mean ± SD and were analyzed by Pearson's chi-square test and Fisher's exact test. Statistical significance was considered at *p < 0.05 and **p < 0.01.

## SUPPLEMENTARY MATERIALS FIGURES


