# Supplementary material for: Glutaredoxin 3 promotes nasopharyngeal carcinoma growth and metastasis via EGFR/Akt pathway and independent of ROS
Source: Oncotarget. 2016 May 18;7(24):37000–12. doi: 10.18632/oncotarget.9454 (PMC5095054; doi:10.18632/oncotarget.9454)
Supplement: Supplementary file 1 [file oncotarget-07-37000-s001.pdf]

Glutaredoxin 3 promotes nasopharyngeal carcinoma growth and metastasis *via* EGFR/Akt pathway and independent of ROS

Supplementary Materials

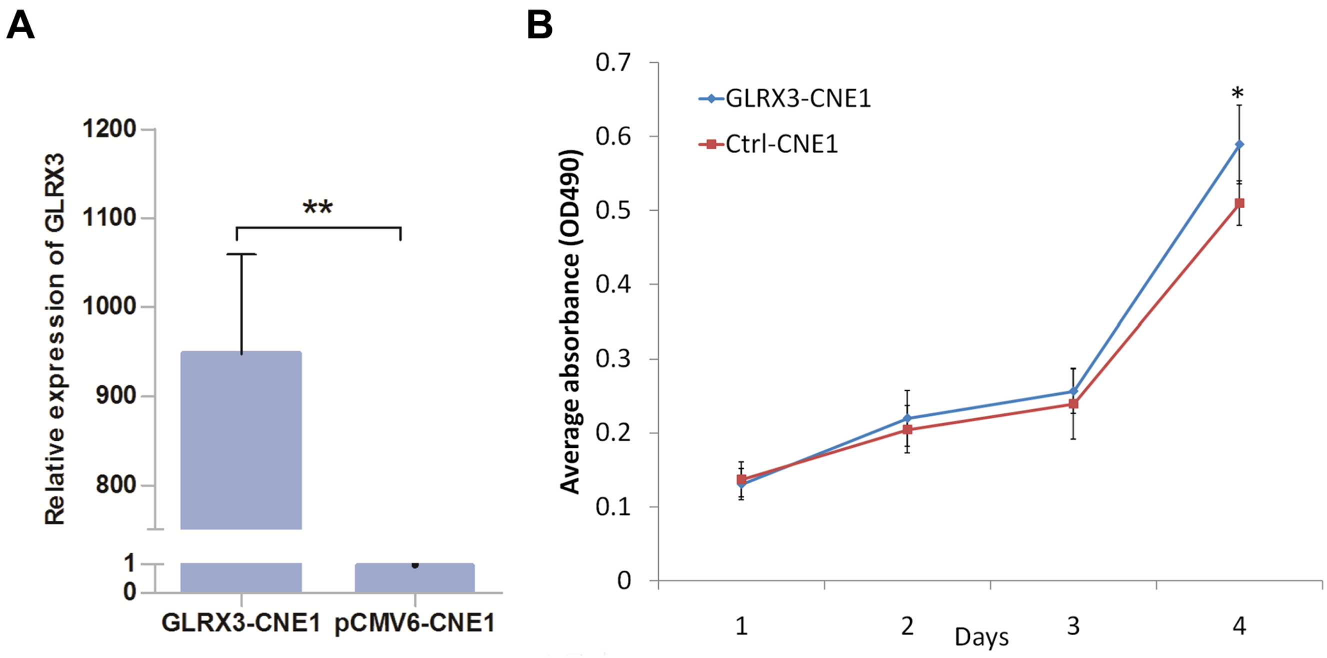

Supplementary Figure S1: Ectopic expression of GLRX3 in CNE1 promotes cell proliferation *in vitro*. (**A**) Confirmation of *GLRX3* transcription in CNE1 cells by real-time PCR. (**B**) CCK8 assay of proliferation ability of *GLRX3*-CNE1 and pCMV6-CNE1 cell lines. Data are mean ± SD of five independent experiments. (*$p < 0.05$).

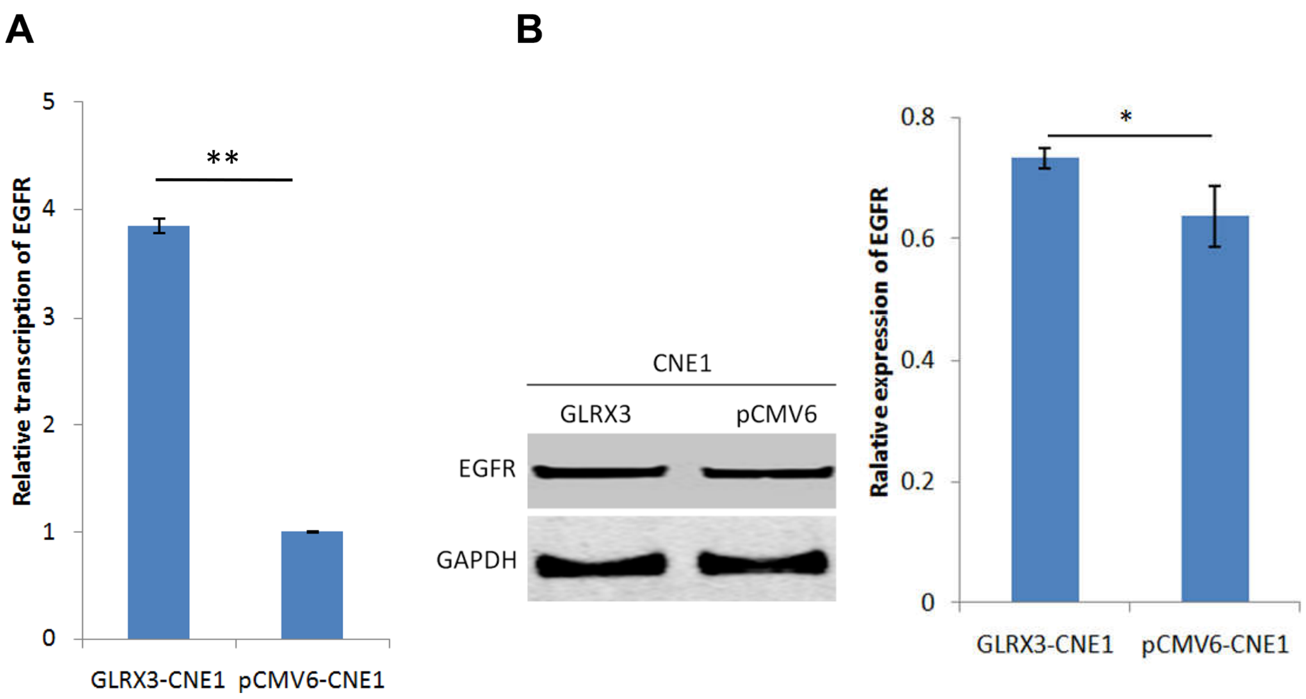

Supplementary Figure S2: GLRX3 stabilizes the expression of EGFR in CNE1 cell line. (**A**) Detection of *EGFR* transcriptional level in *GLRX3*-CNE1 and pCMV6-CNE1 by real-time RT-PCR. (**B**) Analysis of EGFR expression by Western blot. Data are Mean ± SD from three independent experiments. (*$p < 0.05$; **$p < 0.01$).

MATERIALS AND METHODS

Vector construction and transfection

Full-length cDNA from the open reading frame of *GLRX3* inserted in pCMV6-entry plasmid was purchased from Origen Co. (Beijing, China). CNE1 cells were seeded in 6-well plates at a density of 6 × 10^5 cells/well, and transfected with 2 µg pCMV6–entry–*GLRX3* or empty vector pCMV6-entry with X-treme GENE HP DNA Transfection Reagent according to the instruction (Roche Diagnostic, Penzberg, Germany) and incubated for 48 h.
